# Supplementary material for: Co-Creation of Breast Cancer Risk Communication Tools and an Assessment of Risk Factor Awareness: A Qualitative Study of Patients and the Public in India
Source: Cancers (Basel). 2023 May 30;15(11):2973. doi: 10.3390/cancers15112973 (PMC10252022; doi:10.3390/cancers15112973)
Supplement: Supplementary file 1 [file cancers-15-02973-s001.zip › cancers-2330753-supplementary.pdf]

Supplementary Material

File S1. The topic guide used to perform in-depth interviews.

Section S1: Socio-Demographic Information (used for all the groups)

Gender	Male	
	Female	
What is your date of birth?<br><br>If DOB is not known, then give age (in completed years)	Day ……..…. Month…………. Year………..<br><br>……………… years	
In which Indian State / Union Territory were you born?	Assam	
	Delhi	
	Kerala	
	Maharashtra	
	Others	
If other, please name the State / Union Territory		
In which Indian State / Union Territory do you currently reside?	Assam	
	Delhi	
	Kerala	
	Maharashtra	
	Others	
If other, please name the State / Union Territory		
What is your marital status?	Single	
	Married	
	Separated / Divorced	
	Widow / Widower	
	Cohabiting (living with a partner)	
	Other (SPECIFY)	
What is the highest level of education you have completed?	Professional Degree / Postgraduate & above<br>(M.A., M.Sc., PhD, M.Ed. M.B.B.S., B.E., B.Arch.)	
	Graduate (BA/BSc./BCom./Diploma)	
	High School (Class XII pass)	
	Secondary School (Intermediate/(ITI /Class X)	
	Middle School (Class VIII - IX pass)	
	Primary School (Class I to VII)	
	No formal education	
	Others	
If you are married, what is the highest level of education your spouse has completed?	Professional Degree / Post graduate & above	
	Graduate (BA/BSc./BCom./Diploma)	
	High School (Class XII pass)	
	Secondary School (Intermediate/(ITI /Class X)	
	Middle School (Class VIII - IX pass)	

	Primary School (Class I to VII)	
	No formal education	
	Others	
What is your occupational status?	Employed	
	Not employed	
	Retired	
	Homemaker	
	Student	
If you are employed, please specify your occupation		
What is the occupational status of your spouse	Employed	
	Not employed	
	Retired	
	Homemaker	
	Student	
If employed, please specify the occupation		
Could you please provide an estimate of your monthly household income from all sources?	Below 10, 000	
	10, 001 to 30, 000	
	30, 001 to 75, 000	
	75, 001 to 2, 00, 000	
	Above 2 lakh	

Section S2 – Awareness of Breast Cancer and its Risk Factors for Group 1: BC Patients

Knowledge of breast cancer	1. What do you know about breast cancer?<br>2. What are your thoughts on treatment and recovery?<br>3. Are there any ways of detecting breast cancer early?<br>4. Whom did you consult first when you found a breast lump?
Experience of sharing breast cancer diagnosis	1. How did you know/feel when you heard that you had breast cancer?<br>2. Which relatives, if any, did you share this information with?<br>3. What were/are your worries about sharing this information with your family?<br>4. Who would you not want to share such information with?
Awareness of risk factors	1. Are there any habits (drinking alcohol, smoking, exercising), lifestyle choices (number of children, breastfeeding, taking the contraceptive pill), or conditions (excessive weight, diabetes) that can increase the chances of women getting breast cancer?<br>2. Do you think that, in some families, many women may get breast cancer?<br>3. Genes are the basic units of inheritance. Genes are made up of building blocks called DNA. Faults in the DNA code can cause cancer. Have you heard of cancer genes? Have you heard of the *BRCA* gene (the Angelina Jolie gene)?
Risk communication	1. Of the pictures I have shown you regarding factors that reduce the risk of breast cancer, which is the easiest for you to understand?<br>2. Do you have any other suggestions?<br>3. We are going to show you a 3-minute animation to show you the genes responsible for breast cancer and some basic information regarding the mutation of these genes and the hereditary nature of breast cancer. Please let us know if it is easy to understand; if you have any questions, please feel free to ask.<br>4. We are going to read out an infographic strip to detail the genetic testing component of breast cancer. Please feel free to interrupt if you have any

	questions. Let us know whether you have understood it and share if you have any suggestions.<br>5. Of the ways I have shown you (animated video, pictographs, infographics) of communicating risk, which is the easiest for you to understand?<br>6. Do you have any other suggestions?
Participation in research	1. Would you participate in a study to better understand why some women get breast cancer and some do not?<br>2. Would you usually talk to someone other than your doctor before agreeing to take part in a medical study? If yes, who?<br>3. Did you discuss taking part in this study with anyone in the family? If yes, who?
Avenues for seeking health information	1. Where and how do you obtain health information?<br>2. How did you find out so much about breast cancer? (Only if relevant.)<br>3. If we were to give you information on breast cancer and conditions that might increase the chances of developing it, what is your preference from among these media:<br>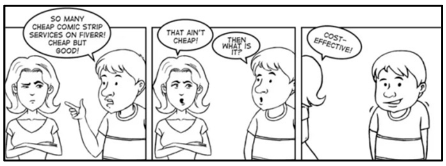COMIC BOOK<br>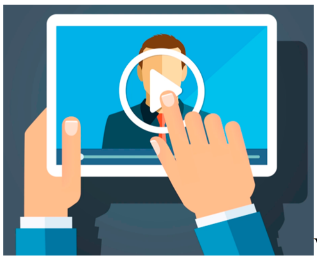VIDEO<br>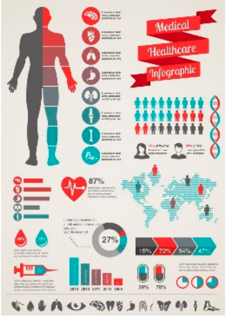POSTER<br>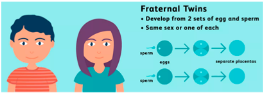ILLUSTRATIONS

Section S3 – Awareness of Breast Cancer and its Risk Factors for Group 2: Relatives

Knowledge of breast cancer	1. What do you know about breast cancer?<br>2. What are your thoughts on treatment and recovery?<br>3. Are there any ways of detecting breast cancer early?<br>4. Where would you go for treatment if you or your close relation had a breast lump?<br>5. Whom would you consult first?<br>6. How expensive do you think breast cancer treatment is?

Experience of sharing BC diagnosis:	1. How did you come to know that your xxx (wife, mother, sister, daughter) had breast cancer?<br>2. How did you feel when you heard it?<br>3. Which members of your family know about her diagnosis?<br>4. Did you ever have a discussion with relatives in your family about her diagnosis? What did you discuss?
Understanding risk factors:	1. Do you know of any habits (e.g., drinking alcohol, smoking, exercising), lifestyle choices (e.g., number of children, breast feeding, taking the contraceptive pill), or conditions (excessive weight, diabetes) that can increase the chance of a women developing breast cancer?<br>2. If one woman has breast cancer, do you think it is possible that others in the family may also develop breast cancer?
Risk communication	1. Of the pictures I have shown you regarding factors that reduce the risk of breast cancer, which is the easiest for you to understand?<br>2. Do you have any other suggestions?<br>3. We are going to show you a 3-minute animation to show you the genes responsible for breast cancer and some basic information regarding the mutation of these genes and the hereditary nature of breast cancer. Please let us know if it is easy to understand; if you have any questions, please feel free to ask.<br>4. We are going to read out an infographic strip to detail the genetic testing component of breast cancer. Please feel free to interrupt if you have any questions. Let us know whether you have understood it and share if you have any suggestions.<br>5. Of the ways I have shown you (animated video, pictographs, infographics) of communicating risk, which is the easiest for you to understand?<br>6. Do you have any other suggestions?
Participation in research	**Female relatives**<br>1. Would you participate in a study to better understand why some women develop breast cancer and some do not?<br>2. Would you usually talk to someone other than your doctor before agreeing to take part in a medical study? If yes, who<br>3. Did you discuss taking part in this study with anyone in the family? If yes, who?<br>**Male relatives**<br>1. Did you discuss taking part in this study with anyone in the family? If yes, who?<br>2. Would you expect women in your family or their doctor to discuss with you / someone else in the family before they join a medical research study?<br>5. What are your thoughts on breast cancer patients joining a study to find out why they might have developed cancer?
Avenues for seeking health information	1. Where and how do you obtain health information?<br>2. How did you find out so much about breast cancer? (Only if relevant.)<br>3. If we were to give you information on breast cancer and conditions that might increase the chances of developing it, what is your preference from among these media:

	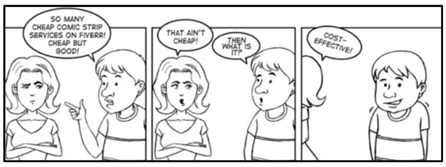 COMIC BOOK<br>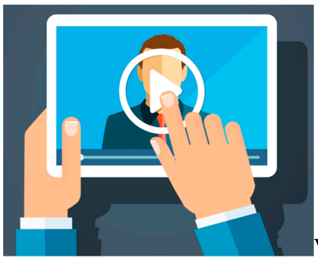 VIDEO<br>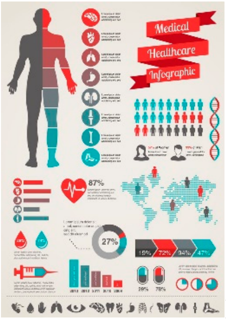 POSTER<br>5. 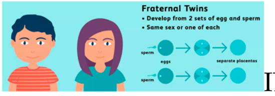 ILLUSTRATIONS

Section S4 – Awareness of Breast Cancer and its Risk Factors for Group 3: Healthy Female Controls

Knowledge of breast cancer:	1. What do you know about cancer? Do you know anything about breast cancer?<br>2. What are your thoughts on cancer treatment and recovery?<br>3. Are there any ways of detecting cancer early, especially breast cancer?<br>4. Where would you go for treatment if you or your close relation had a breast lump?<br>5. Whom would you consult first?<br>6. How expensive do you think breast cancer treatment is? (Type box.)
Sharing BC diagnosis	1. Do you think that, if somebody is diagnosed with cancer, they should share this information with others?<br>2. Should they tell their relatives?<br>3. How would this help?<br>4. Do you know about people who do not share their cancer diagnosis with relatives? Why do you think that is?
Understanding risk factors	1. Do you know of anything that increases the chance of developing cancer?<br>2. With regard to breast cancer, do you know of any habits (e.g., drinking alcohol, smoking, exercising), lifestyle choices (e.g., number of children, breast feeding, taking the contraceptive pill), or conditions (excessive weight, diabetes) that can increase the chance of a women developing breast cancer?<br>3. Do you know if cancer can occur in many people from one family? Why do you think this happens?
Risk communication	1. Of the pictures I have shown you regarding factors reducing the risk of breast cancer, which is the easiest for you to understand?

	2. Do you have any other suggestions?<br>3. We are going to show you a 3-minute animation to show you the genes responsible for breast cancer and some basic information regarding the mutation of these genes and the hereditary nature of breast cancer. Please let us know if it is easy to understand; if you have any questions, please feel free to ask.<br>4. We are going to read out an infographic strip to detail the genetic testing component of breast cancer. Please feel free to interrupt if you have any questions. Let us know whether you have understood it and share if you have any suggestions.<br>5. Of the ways I have shown you (animated video, pictographs, infographics) of communicating risk, which is the easiest for you to understand?<br>6. Do you have any other suggestions?
Participation in research:	1. Would many of your patients / their relatives participate in a study to better understand breast cancer risk?<br>2. Do you think unaffected healthy people in the wider population would be willing to take part?<br>3. Do they usually talk to someone in the family before agreeing to take part? If yes, who?<br>4. Do your female patients usually require permission for others in the family before they join a study? If yes, who (clinicians only)?<br>5. What are your thoughts on studies to better understand why some Indian women get breast cancer and some do not?<br>Would you support recruitment to such a study? How would you do so?
Avenues for seeking health information	1. Where and how do patients/the public obtain health information?<br>2. What is your preference from among these media (provide examples so that the individual can choose) with regard to developing study information?<br>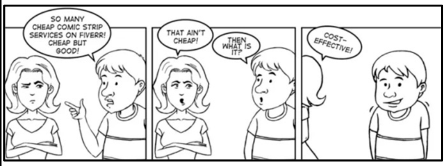 COMIC BOOK<br>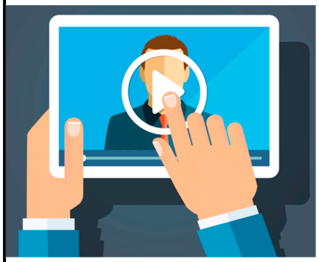 VIDEO<br>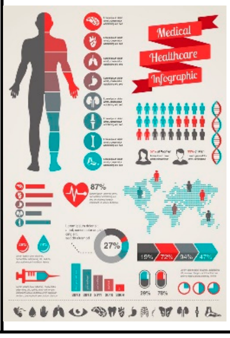 POSTER

<table>
<tr><td></td><td>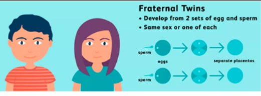

ILLUSTRATIONS</td></tr>
</table>

Section S5 – Awareness of Breast Cancer and its Risk Factors for Group 4: Individuals from cancer charities/ clinical teams

Knowledge of breast cancer	1. How much do your patients/the public know about breast cancer, its treatment, and the chances of survival?<br>2. How aware are patients/their families/the public that it can be picked up early?<br>3. Do many women participate in breast cancer screening/ breast self-examination?<br>4. Do any women opt for risk-reducing surgery (the removal of normal breasts) or take drugs such as tamoxifen to reduce breast cancer risk?
Sharing BC diagnosis	1. Do women tell their relatives about their breast cancer diagnosis?<br>2. Who do they tell?<br>3. If patients do not share their cancer diagnosis with wider family, why do you think that is?
Understanding risk factors	1. Are patients/the public aware of breast cancer risk factors?<br>2. Do they know about the genetic predisposition to cancer in some families?<br>3. Are they willing to undergo genetic testing?<br>4. What are their main concerns?
Risk communication	1. Of the pictures I have shown you regarding factors reducing the risk of breast cancer, which is the easiest for you to understand?<br>2. Do you have any other suggestions?<br>3. We are going to show you a 3-minute animation to show you the genes responsible for breast cancer and some basic information regarding the mutation of these genes and the hereditary nature of breast cancer. Please let us know if it is easy to understand; if you have any questions, please feel free to ask.<br>4. We are going to read out an infographic strip to detail the genetic testing component of breast cancer. Please feel free to interrupt if you have any questions. Also, let us know whether you have understood it and share if you have any suggestions.<br>5. Of the ways I have shown you (animated video, pictographs, infographics) of communicating risk, which is the easiest for you to understand?<br>5. Do you have any other suggestions?
Participation in research:	1. Would many of your patients / their relatives participate in a study to better understand breast cancer risk?<br>2. Do you think unaffected healthy people in the wider population would be willing to take part?<br>3. Do they usually talk to someone in the family before agreeing to take part? If yes, who?<br>4. Do your female patients usually require permission from others in the family before they join a study? If yes, who (clinicians only)?

	5. What are your thoughts on studies to better understand why some Indian women get breast cancer and some do not?<br>6. Would you support recruitment to such a study? How so?
Avenues for seeking health information	1. Where and how do patients/the public obtain health information?<br>2. What is your preference from among these media (provide examples so that the individual can choose) with regard to developing study information?<br>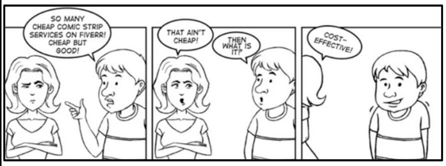 COMIC BOOK<br>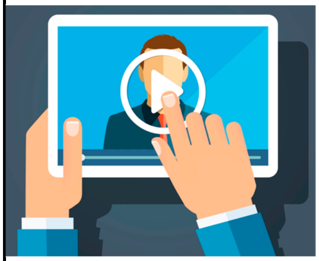 VIDEO<br>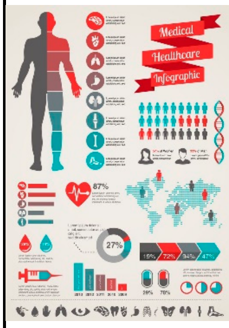 POSTER<br>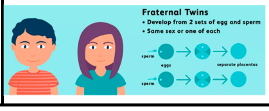 ILLUSTRATIONS
